# Platelet distribution width as an useful indicator of influenza severity in children

**DOI:** 10.1186/s12879-023-08890-w

**Published:** 2024-01-02

**Authors:** Seyin Zou, Siti Hasmah Mohtar, Roshani Othman, Rodiah Mohd Hassan, Kun Liang, Da Lei, Bangming Xu

**Affiliations:** 1 Department of Laboratory Medicine, Guangdong Second Provincial General Hospital, Guangzhou, 510317 China; 2 Department of Science and Biotechnology, Faculty of Engineering and Life Sciences, Universiti Selangor, Bestari Jaya Campus, Bestari Jaya, Selangor Darul Ehsan 45600 Malaysia; 3 Guangdong Medical University, Dongguan, 523000 China

**Keywords:** Influenza, Platelet parameters, Platelet distribution width, Severity assessment, Neutrophil-to-lymphocyte ratio

## Abstract

**Purpose:**

The present study aims to investigate the potential of platelet distribution width as an useful parameter to assess the severity of influenza in children.

**Methods:**

Baseline characteristics and laboratory results were collected and analyzed. Receiver operating characteristic (ROC) curve analysis was used to joint detection of inflammatory markers for influenza positive children, and the scatter-dot plots were used to compare the differences between severe and non-severe group.

**Results:**

Influenza B positive children had more bronchitis and pneumonia (*P* < 0.05), influenza A infected children had more other serious symptoms (*P* = 0.007). Neutrophil count, lymphocyte count, neutrophil-to-lymphocyte ratio (NLR), and platelet parameters performed differently among < 4 years and ≥ 4 years children with influenza. Combined detection of platelet parameters and other indicators could better separate healthy children from influenza infected children than single indicator detection. The levels of platelet distribution width of children with severe influenza (A and B) infection was significantly dropped, compared with non-severe group (*P* < 0.05)*.*

**Conclusions:**

Platelet distribution width could be a very useful and economic indicator in distinction and severity assessment for children with influenza.

## Introduction

Influenza is an acute viral respiratory disease caused by influenza A and B viruses. Annual seasonal influenza epidemics of variable severity lead to morbidity and mortality worldwide [1–3]. Influenza is one of the commonest causes of acute respiratory illness and loss of school days in children. Household-based and community-based studies in different countries have shown that influenza virus infections and illness rates are highest in children [2]. The infection is usually self-limiting, although some individuals particularly young children have a higher risk of complications, such as pneumonia, septic shock and multiple organ dysfunction, resulting in hospital admissions, or in hospital deaths [4]. It is worth noting that most individuals with influenza disease, both children and adults, that required intensive care, had no underlying conditions [5, 6]. Therefore, biomarkers that help with early diagnosis are essential to detect severe influenza infection in children.

Platelets (PLTs), fragments of megakaryocyte cytoplasm, play a central role in pathological thrombosis [7]. In addition, platelets significantly contribute to the inflammatory reactions and immune response in various types of infections including influenza [8, 9]. Previous studies indicate that platelet parameters are associated with the inflammatory response and severity of many infectious diseases [10–13]. Platelet parameters consist of PLT count, platelet distribution width (PDW), mean platelet volume (MPV), and plateletcrit (PCT), obtained by automated cell counters. Yu et al. showed that PLT count was an independent predictor of severe hemorrhagic fever with renal syndrome (HFRS) caused by Hanta virus [11], Barrett et al. showed that MPV associate with increased critical illness and all-cause mortality among hospitalized COVID-19 patients [12]. Additionally, PDW has been reported to be a widely applied and reliable marker of platelet activation and have recently been shown to be an independent risk factor of postoperative pneumonia in patients with type A acute aortic dissection (AAAD) [10], as well as an useful indicator for patients with severe burns [13]. However, the role of these parameters in influenza has been rarely investigated. There are very few reports about their clinical values to distinguish non-severe from severe influenza in children. The aim of the current study, therefore, was to investigate whether platelet parameters would be useful in distinction and evaluating the severity of influenza virus infection.

## Methods

### Patients and inclusion criteria

A retrospective study was conducted by analyzing data collected from children confirmed influenza, first-visit outpatients at the Guangdong Second Provincial General Hospital from January 1, 2017 to December 31, 2020. The study complied with the Declaration of Helsinki, and the Ethics Committee of Guangdong Second Provincial General Hospital approved the study protocol. We obtained informed consent from all participants and their legal guardians involved in our study. Participants enrolled in the study were given written informed consent. A total of 636 cases were enrolled: 64 healthy controls, 424 influenza A children and 148 influenza B children. The diagnosis of influenza A and B were confirmed by laboratory test for influenza virus antigens from the nasopharyngeal swab samples showing positive result by the colloidal gold method with a very high specificities (> 98%) [14, 15]. Meanwhile, children combining with other infections or having undergone antiviral therapy were excluded. All the patients enrolled in this study underwent routine blood tests and the detection of influenza virus antigens within 48 h of the onset of fever or acute respiratory symptom. According to the clinical practice guidelines for the diagnosis and management of children with influenza (2020) [15, 16], Both children with influenza A and B were divided into non-severe and severe group. ‘Non-severe’ group was defined as influenza infected children with mild clinical symptoms, fever, cough, laryngopharyngitis tracheitis, bronchitis, gastroenteritis or other influenza symptoms; ‘severe’ group was defined as influenza infected children occurring one or more of following situations: pneumonia, agitation, unresponsiveness, convulsions, somnolence, dyspnea, vomiting and diarrhea with dehydration or electrolyte imbalance. Meanwhile, 64 healthy subjects (35 < 4 years and 29 ≥ 4 years), who came to the hospital for a health care or a routine examination, were enrolled as a healthy control group after being proved to have normal laboratory indicator levels, did not have any respiratory or other infection symptoms.

### Instruments and reagents

The neutrophil count, lymphocyte count, platelet count, mean platelet volume (MPV), platelet distribution width (PDW), and plateletcrit (PCT) were determined with a BC-5390 automatic hematology analyzer and associated reagents (Shenzhen Mindray Bio-Medical Electronics Co. Ltd., China), according to the manufacture’s instruction. The equipment was maintained and calibrated according to the requirements. Internal quality control and external quality assessment were performed.

### Data collection

For each patient, all laboratory tests were conducted at admission and the following data were collected: clinical characteristics, laboratory results including complete blood count, and chest X-ray. Patient data was obtained from hospital computer database system and patients’ medical records.

### Statistical analyses

Data were analyzed using the IBM SPSS Statistics 17.0, and data were expressed as median ± standard deviation (SD). The Independent sample t-test was used to compare two independent groups, categorical variables were analyzed with chi-square test. The Games-Howell one-way ANOVA was used to compare multiple groups, the combined detection of laboratory indicators was analyzed with the receiver operating characteristic curve (ROC). All tests were two-sided and a *P* < 0.05 was considered statistically significant.

## Results

### Baseline characteristics of children with influenza

**Fig. 1:**
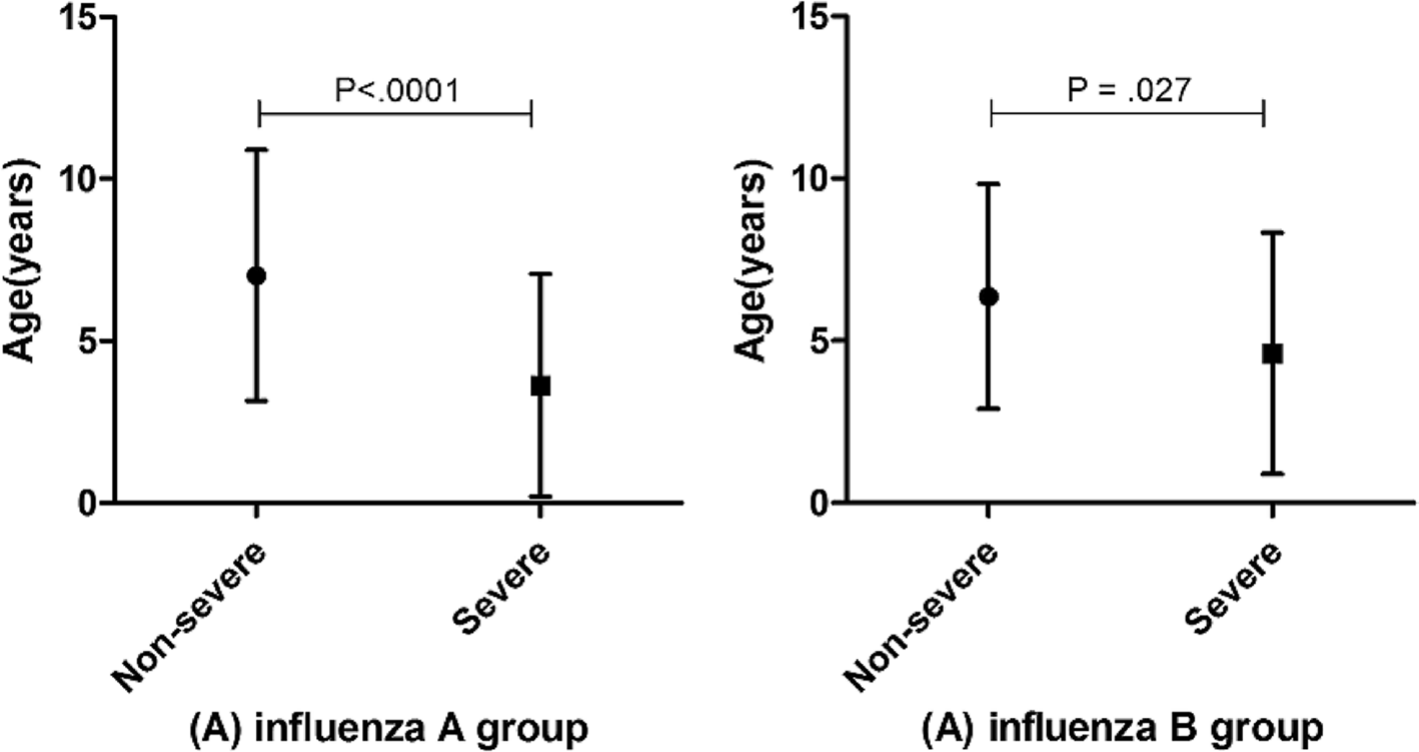
Comparison of age for children with non-severe and severe Influenza infection. Error bars indicate the mean and standard deviation of age of non-severe group and severe group for children with Influenza A (**A**) and Influenza B (**B**)

A total of 572 children with influenza were included in this study, 424 of those with influenza A and the remaining 148 with influenza B. There was no statistical difference in the sex ratio between the influenza A group and influenza B group (*P* = 0.706). The mean age was slightly higher among children with influenza A than those with influenza B, although the difference was not significant (*P* = 0.325). Table 1 shows the signs and symptoms of influenza in the children at presentation. Their main symptoms at admission were fever and laryngopharyngitis for either children with influenza A or B, other influenza symptoms was more common in children with influenza A (*P* = 0.003) and bronchitis occurred more frequently among children with influenza B (*P* < 0.0001). Other serious complications were recorded more frequently among influenza A positive children (*P* = 0.0007) while more cases developed pneumonia for children with influenza B (*P* = 0.041) (Table 1). Besides, independently of the influenza virus, mean age in severe patients was lower compared to the non-severe group (*P* < 0.05) (Fig. 1).

**Table 1:** Baseline children with influenza A and B

Characteristic	Influenza A(*n* = 424)	Influenza B(*n* = 148)	t/X2	*P* value
age (median ± SD)	6.42 ± 3.99	6.07 ± 3.56	0.987	0.325
sex (male/female)	259/165	93/55	0.142	0.706
Clinical symptoms				
Laryngopharyngitis, no.(%)	273(64.4)	96(64.9)	0.011	0.917
Fever,no.(%)	330(77.8)	106(71.6)	2.33	0.127
Bronchitis,no.(%)	107(25.2)	60(40.5)	12.43	< 0.0001
Cough,no.(%)	171(40.3)	48(32.4)	0.003	0.956
Gastroenteritis, no.(%)	86(20.3)	30(20.3)	0	0.997
Other Influenza symptoms,no.(%)	184(43.4)	44(29.7)	8.548	0.003
Amygdalitis,no.(%)	8(1.9)	7(4.7)	–	0.075
Pneumonia,no.(%)	40(9.4)	23(15.5)	4.174	0.041
Other serious symptoms,no.(%)	36(8.5)	3(2.4)	7.214	0.007

t: comparison in age between two groups; X2: comparison of categorical variables. Other serious symptoms included dyspnea, unresponsiveness, agitation, somnolence, convulsions, vomiting and diarrhea with dehydration or electrolyte imbalance

### Laboratory indicators of children with influenza

Influenza-infected children (A or B viruses) under 4 years old presented significantly higher neutrophil count, NLR (neutrophil-to-lymphocyte ratio) and lower PDW compared to healthy individuals. However, no differences were found when comparing influenza A versus influenza B infected children of the same age (*P* > 0.05). Additionally, influenza A groups’ MPV was significantly lower than that of healthy controls (*P* = 0.021) (Table 2). Among children over 4 years old, significant increase in neutrophil count, NLR, MPV, PDW and decrease in lymphocyte count, platelet count, PCT were observed in children with influenza (A and B) compared to healthy control group (*P* < 0.05). Moreover, the neutrophil count, NLR, platelet count, and PCT were significantly higher in children with influenza A than those with influenza B (*P* < 0.05) (Table 3).

**Table 2:** Laboratory indicators of children(< 4 years) with influenza A and B (median ± SD)

Indicators	Control group	Influenza A group	Influenza B group	*P* value a	*P* value b
	*n* = 35	*n* = 132	*n* = 40		
Neutrophil count, × 10^9^/L	3.62 ± 1.23	5.83 ± 3.60	5.37 ± 3.34	< 0.0001	0.009
Lymphocyte count, × 10^9^/L	2.11 ± 0.56	3.01 ± 2.73	2.65 ± 1.87	0.132	0.944
NLR	1.77 ± 0.54	4.07 ± 4.41	3.27 ± 3.78	< 0.0001	0.045
Platelet count, × 10^9^/L	270.66 ± 42.24	263.43 ± 101.93	248.03 ± 103.56	0.801	0.42
MPV, fl	9.15 ± 1.03	8.74 ± 0.90	8.81 ± 0.78	0.021	0.309
PCT, %	0.25 ± 0.04	0.23 ± 0.09	0.22 ± 0.09	0.85	0.407
PDW, %	15.74 ± 0.26	15.95 ± 0.53	15.93 ± 0.45	0.004	0.027

*Abbreviations: NLR* neutrophil-to-lymphocyte ratio, *MPV* mean platelet volume, *PCT* plateletcrit, *PDW* platelet distribution width

^a^ comparison between Influenza A and Control group

^b^ comparison between Influenza B and Control group

**Table 3:** Laboratory indicators of children(≥4 years) with influenza (mean ± SD)

Indicators	Control group *n* = 29	Influenza A group *n* = 292	Influenza B group *n* = 108	*P* value ^a^	*P* value ^b^	*P* value ^c^
Neutrophil count, ×109/L	3.57 ± 1.17	6.89 ± 3.03	4.65 ± 2.40	< 0.0001	0.003	< 0.0001
Lymphocyte count,× 109/L	2.37 ± 0.50	1.41 ± 0.73	1.50 ± 0.73	< 0.0001	< 0.0001	0.579
NLR	1.53 ± 0.47	6.62 ± 6.04	4.03 ± 3.84	< 0.0001	< 0.0001	< 0.0001
Platelet count, × 109/L	292.10 ± 54.71	246.33 ± 63.43	200.96 ± 54.82	< 0.0001	< 0.0001	< 0.0001
MPV, fl	8.39 ± 0.79	9.00 ± 0.83	9.04 ± 0.95	0.001	0.001	0.922
PCT, %	0.244 ± 0.04	0.220 ± 0.05	0.178 ± 0.04	0.026	< 0.0001	< 0.0001
PDW, %	15.60 ± 0.26	16.20 ± 0.62	16.21 ± 0.63	< 0.0001	< 0.0001	0.983

*Abbreviations: NLR* neutrophil-to-lymphocyte ratio, *MPV* mean platelet volume, *PCT* plateletcrit, *PDW* platelet distribution width

^a^ comparison between Influenza A and Contro l

^b^ comparison between Influenza B and Control group

^c^ comparison between Influenza A and B group

### Joint detection of indicators in children with influenza

**Fig. 2:**
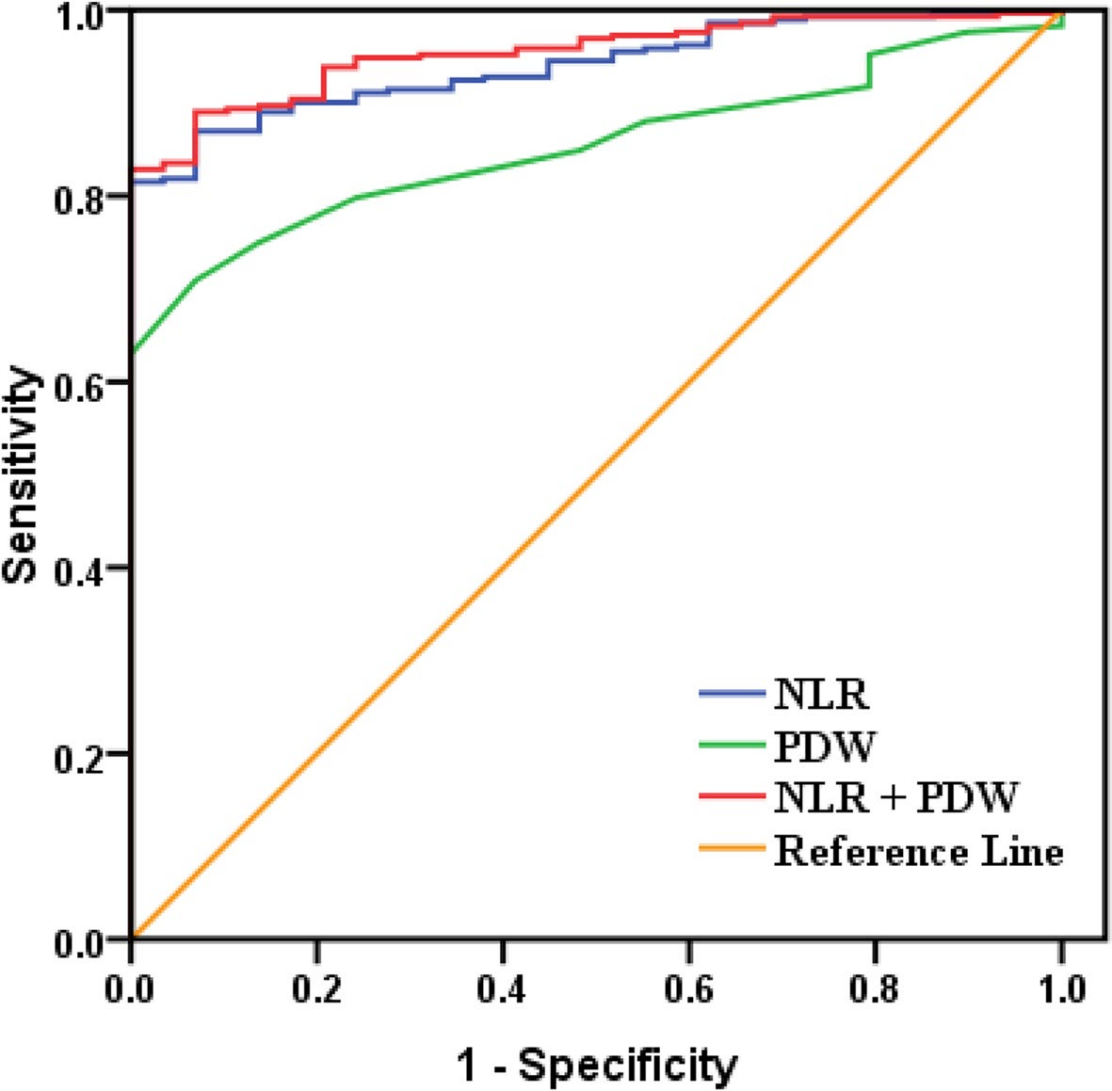
ROC curve of PDW and NLR for children with Influenza A(≥4 years). When the detection of PDW alone, the area under the ROC curve was 0.853 (95% CI: 0.808–0.898; *P* < 0.05). When the detection of NLR alone, the area under the ROC curve was 0.940(95% CI: 0.913–0.968; *P* < 0.05). When the combined detection of them, the area under the ROC curve was 0.954 (95% CI: 0.930–0.978; *P* < 0.05)

**Fig. 3:**
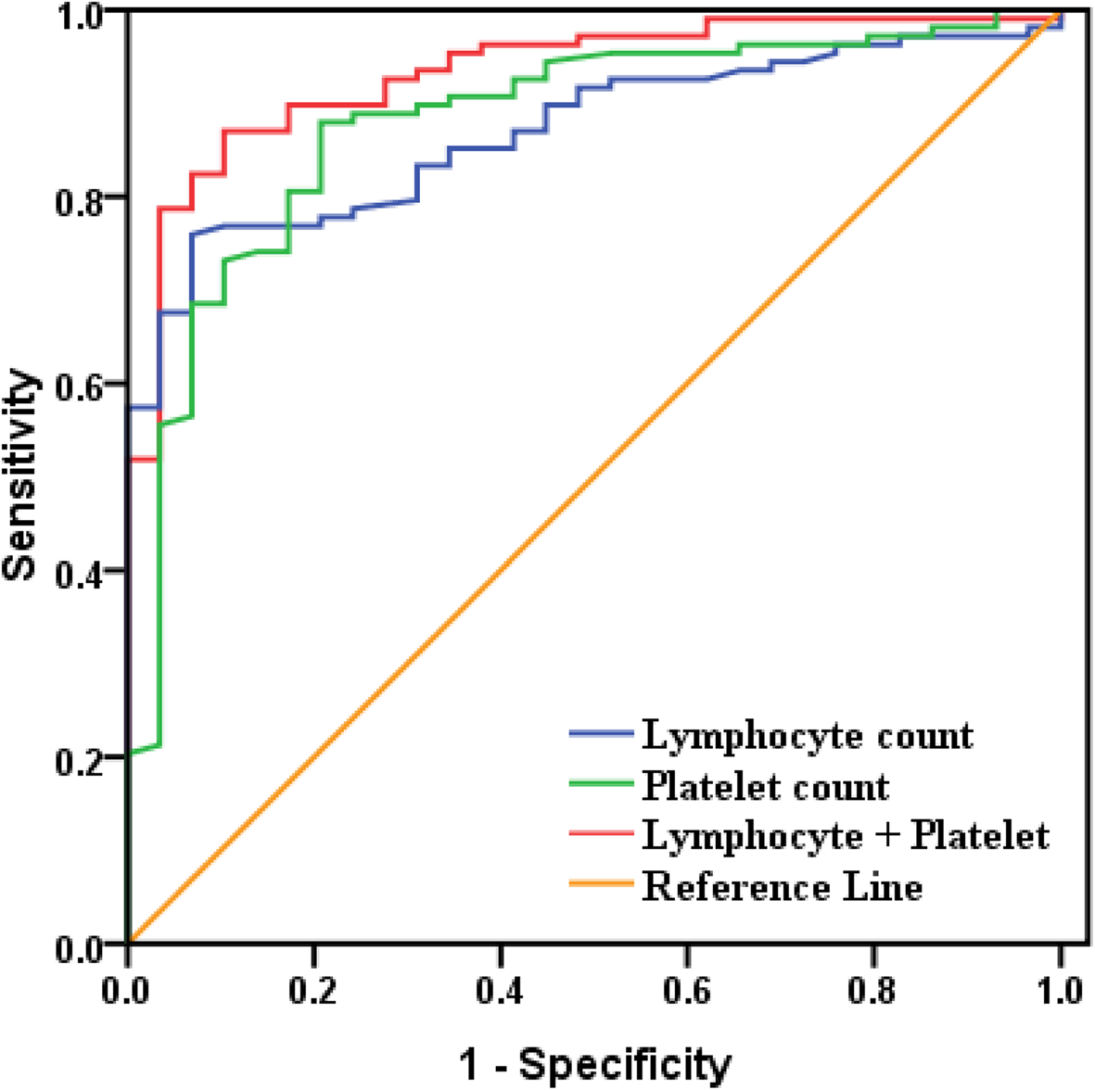
ROC curve of Platelet count and Lymphocyte count for children with Influenza B(≥4 years). When the detection of Lymphocyte count alone, the area under the ROC curve was 0.870(95% CI: 0.811–0.929; *P* < 0.05). When the detection of Platelet count alone, the area under the ROC curve was 0.879 (95% CI: 0.810–0.949; *P* < 0.05). When the combined detection of them, the area under the ROC curve was 0.933 (95% CI: 0.888–0.978; *P* < 0.05)

**Fig. 4:**
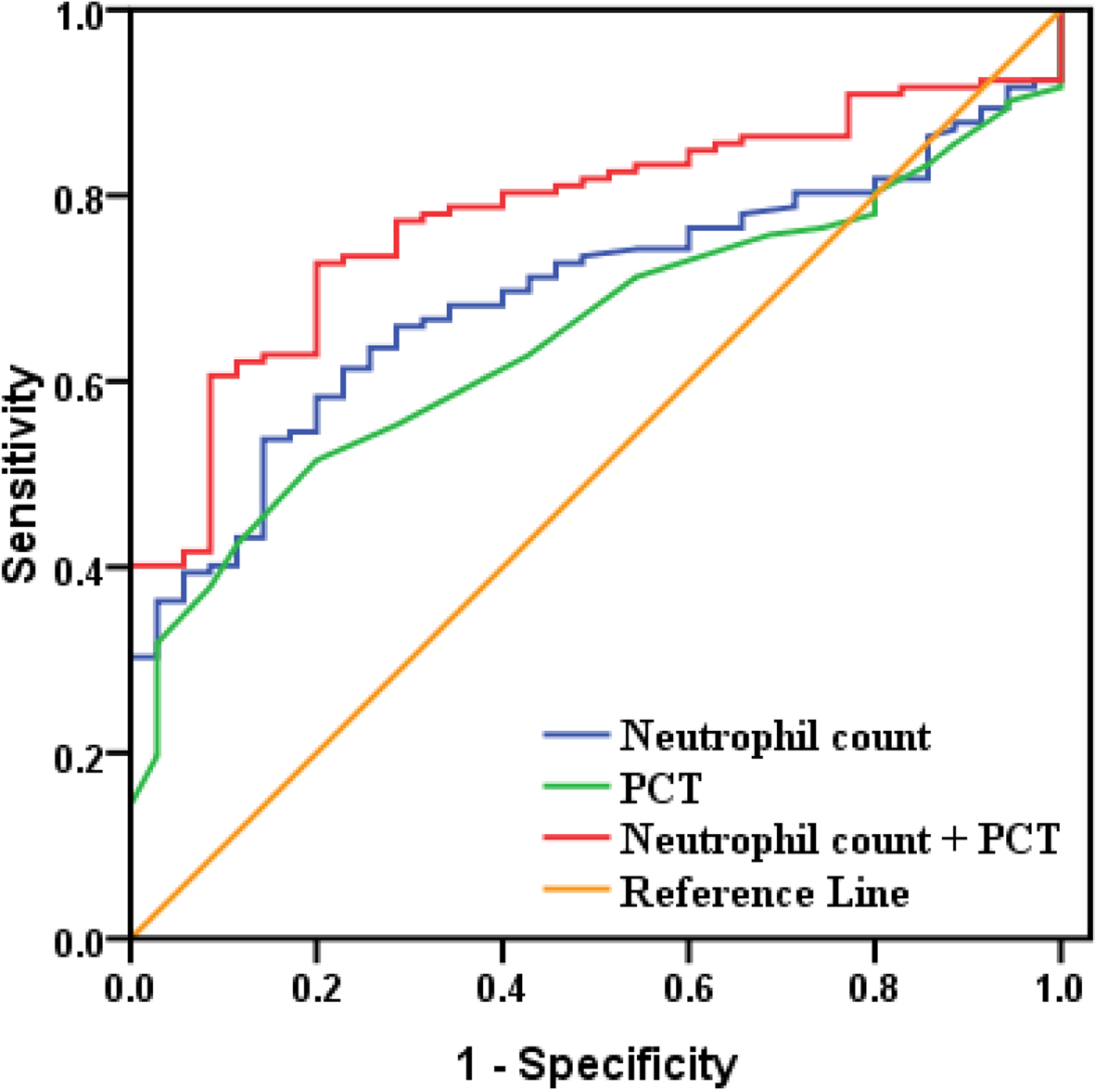
ROC curve of Neutrophil count and PCT for children with Influenza A(< 4 years). When the detection of PCT alone, the area under the ROC curve was 0.649 (95% CI: 0.564–0.734; *P* < 0.05). When the detection of Neutrophil count alone, the area under the ROC curve was 0.692(95% CI: 0.611–0.773; *P* < 0.05). When the combined detection of them, the area under the ROC curve was 0.779 (95% CI: 0.707–0.851; *P* < 0.05)

**Fig. 5:**
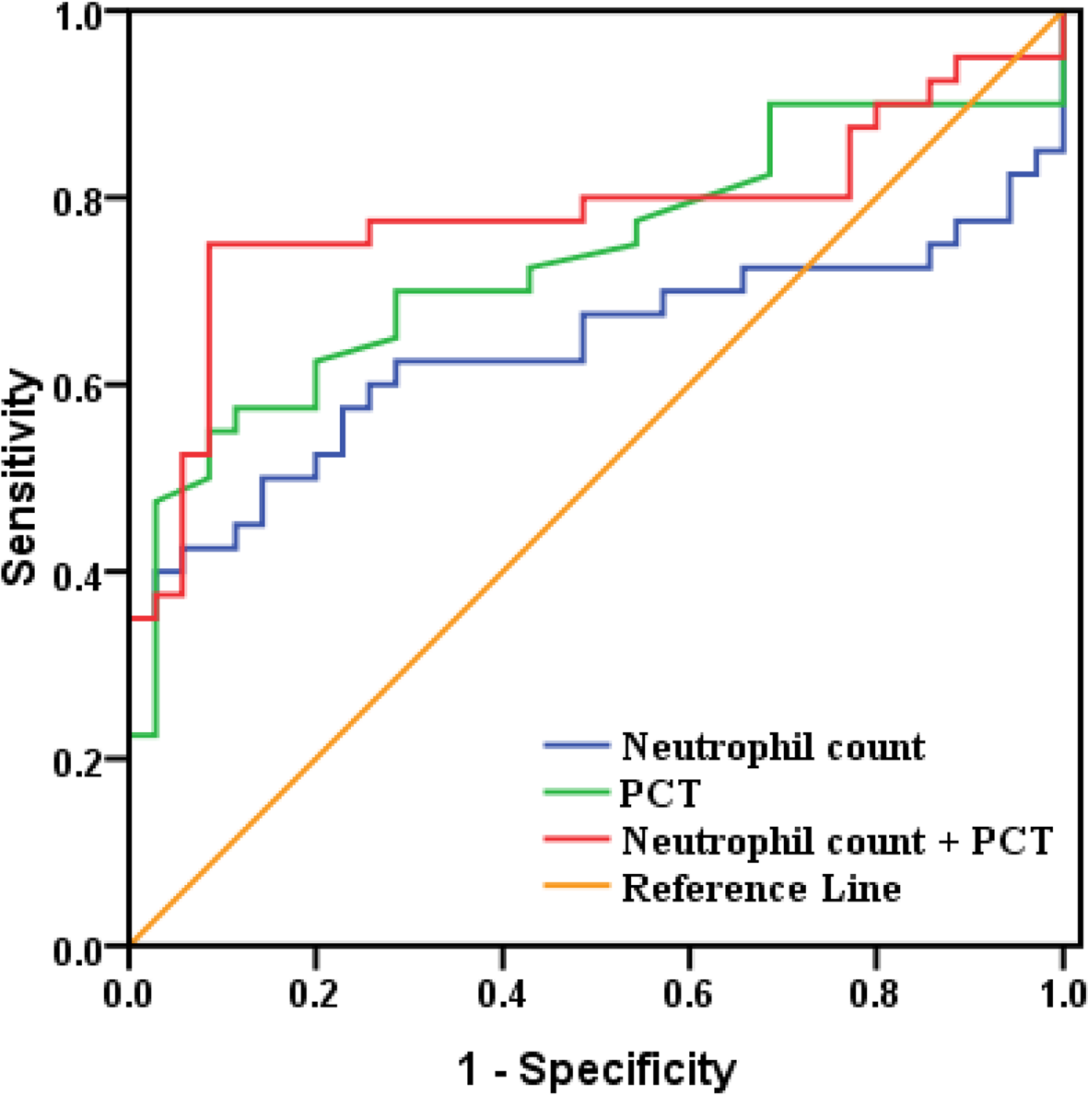
ROC curve of Neutrophil count and PCT for children with Influenza B(< 4 years). When the detection of PCT alone, the area under the ROC curve was 0.735 (95% CI: 0.619–0.851; *P* < 0.05). When the detection of Neutrophil count alone, the area under the ROC curve was 0.637(95% CI: 0.505–0.770; *P* < 0.05). When the combined detection of them, the area under the ROC curve was 0.781 (95% CI: 0.669–0.894; *P* < 0.05)

Based on the detection of virus antigen for diagnosing influenza A and B, the area under curve (AUC) of lymphocyte count, neutrophil count, NLR, platelet count, MPV, PCT, and PDW in influenza A group were 0.437, 0.692, 0.680, 0.589, 0.613, 0.649, 0.592(< 4 years), and 0.871, 0.868, 0.940, 0.737, 0.721, 0.670, 0.853(≥ 4 years group), respectively. For influenza B group, the AUC of lymphocyte count, neutrophil count, NLR, platelet count, MPV, PCT, and PDW were 0.538, 0.637, 0.507, 0.683, 0.578, 0.735, 0.619 (< 4 years group) and 0.87, 0.629, 0.831, 0.879, 0.712, 0.865, 0.821 (≥ 4 years group), respectively. The ROC curve analysis in children over 4 years old showed that the combined of NLR and PDW for distinguishing healthy children from children with influenza A was better than detection of NLR or PDW alone (Fig. 2). And compared with lymphocyte count and platelet count, the ROC curve analysis showed that the combined of both for distinguishing healthy children from children with influenza B was better than detection of lymphocyte count or platelet count alone (Fig. 3). Interestingly, among children aged under 4 years, compared to neutrophil count and PCT, the ROC curve analysis showed that joint detection of neutrophil count and PCT could better separate healthy children from infected children than single indicator detection, no matter which influenza virus were infected with (Figs. 4 and 5).

### Comparison of laboratory indicators among severe and non-severe children with influenza

**Fig. 6:**
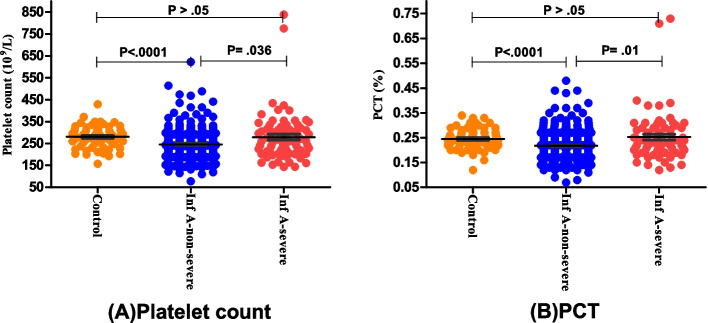
Comparison of laboratory indicators among children with Influenza A. Scatter-dot plots showing the levels of Platelet count (**A**) and PCT (**B**) among control group, non-severe group, and severe group. Error bars in the scatter-dot plots indicate the mean and standard deviation. Inf:Influenza

**Fig. 7:**
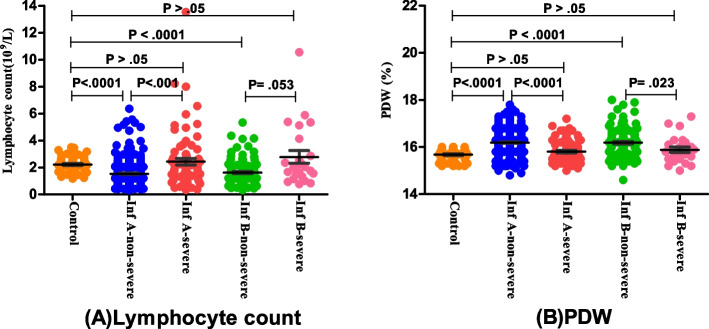
Comparison of laboratory indicators among children with Influenza. Scatter-dot plots showing the levels of Lymphoctye count (**A**) and PDW (**B**) among control group, non-severe group, and severe group. Error bars in the scatter-dot plots indicate the mean and standard deviation. Inf:Influenza

Four indicators including Lymphoctye count, Platelet count, PCT, and PDW in children with influenza A were all significantly different in the non-severe group compared with those in the severe group, as well as with heathy group(*P* < 0.05); but there were no statistical differences between the severe influenza A group and control group. In addition, the level of PDW in children with influenza B also showed a significant lower in the severe group, compared with the non-severe group (*P* = 0.023). However, the neutrophil count, NLR, and MPV were not statistically different among non-severe and severe children both in influenza A and B group (Table 4, Figs. 6 and 7).

**Table 4:** Comparison of laboratory indicators among children with influenza (mean ± SD)

Indicators	Control group	Influenza A group			Influenza B group		
		Non-severe	Severe	*P* value	Non-severe	Severe	*P* value
n	64	349	75		124	24	
Neutrophil count,×109/L	3.60 ± 1.19	6.45 ± 2.90	7.08 ± 4.52	0.777	4.79 ± 2.59	5.12 ± 3.22	0.989
Lymphocyte count,× 109/L	2.22 ± 0.55	1.54 ± 0.91	2.43 ± 2.09	0.004	1.62 ± 0.84	2.78 ± 2.27	0.053
NLR	1.11 ± 0.91	5.88 ± 4.96	5.57 ± 8.37	0.998	4.01 ± 3.99	2.91 ± 2.72	0.231
Platelet count, × 109/L	280.38 ± 49.08	245.68 ± 67.02	279.4 ± 111.8	0.036	206.4 ± 59.3	251.3 ± 120.2	0.195
MPV, fl	8.81 ± 0.99	8.89 ± 0.84	9.08 ± 0.92	0.186	9.00 ± 0.94	8.85 ± 0.77	1
PCT,%	0.245 ± 0.041	0.217 ± 0.056	0.252 ± 0.099	0.028	0.183 ± 0.044	0.223 ± 0.108	0.404
PDW, %	15.68 ± 0.26	16.19 ± 0.60	15.80 ± 0.49	< 0.0001	16.18 ± 0.59	15.88 ± 0.58	0.023

Abbreviations: *NLR* neutrophil-to-lymphocyte ratio, *MPV* mean platelet volume, *PCT* plateletcrit, *PDW* platelet distribution width

## Discussion

Influenza primarily affects the respiratory system and represents one of the most frequent causes of acute upper respiratory tract infections during the winter season. During 4 non-pandemic influenza seasons (2017–2020), about 424 influenza A and 148 influenza B cases were identified in this study. The prevalence rate of influenza A was much higher than that of influenza B. This is consistent with our previous findings and other research that influenza A had more ability to cause pandemics than influenza B [17, 18]. Additionally, being a little different from report of other research group [19], this study showed that influenza B positive children were more prone to be infected in the lower respiratory tract (bronchitis and pneumonia), the possible reasons for this difference are uncertain and require further study. On the other hand, influenza A was more likely to cause other system symptoms in addition to respiratory symptoms. It may be because influenza A virus is the most virulent influenza virus type and leads to the most severe disease outcomes [20]. Furthermore, we found that young age, as already reported, is an important risk factor for the severe influenza infection [21].

It is very difficult to diagnose influenza in young children based on its clinical symptoms alone, as no existing specific signs [22]. Therefore, laboratory indicators play an essential role in the early accurate diagnosis and severity assessment. In view of the opposite change trend of lymphocyte and neutrophil with age, and platelet parameters may be partly age-dependent [23, 24], the subjects were divided into the < 4 years group and ≥ 4 years group. We analyzed the results of laboratory indicators and found that lymphocyte count, neutrophil count, NLR, Platelet count, MPV, PCT, and PDW all showed a significant difference for children(≥ 4 years) with influenza A and B when compared to healthy individuals, while different situations were present among children under 4 years old. The different performance between these two groups may be due to the fact that children under 4 years old are an immunologically naïve population, indicating less immune response and inflammatory reaction [25, 26]. This pattern seemed to reflect the development of acquired and adaptive immune system in children [27]. These results indicated that lymphocyte count, neutrophil count, NLR, Platelet count, MPV, PCT, and PDW could be used as indicators for distinguishing children(≥ 4 years) with influenza from health, while neutrophil count and PDW could be used as biomarkers to differentiate influenza infection in children younger than 4 years old. Lymphocyte count and Neutrophil count are convenient and quick index of inflammation detections in laboratory examination, they are used in the diagnosis, treatment and prognosis evaluation of many disease [28, 29]. Platelet parameters, as newer, easily accessible, and inexpensive biomarkers had been reported to increase the accuracy of the diagnosis of many other infections [30–32]. We found that the joint detection of platelet parameters and other inflammatory indicators showed better performance than detection of single indicator.

According to the clinical practice guidelines for the diagnosis and management of children with influenza [15, 16], we divided children into a non-severe and severe group. The findings from this study show that the inflammatory indicators involved performed very differently between non-severe and severe group in children with influenza A and B. Indeed, there are more inflammation indicators that displayed significant differences between the non-severe and severe group in influenza A. According to the previous research, influenza A disease is more severe than influenza B disease, with a more intense inflammatory response that reflects in an increase in systemic inflammatory markers [19, 33, 34]. Our findings are consistent with these prior findings. Moreover, we found that the platelet count was significantly decreased and PDW showed a marked increase in the non-severe group, especially for children with influenza A. Many studies have confirmed that many receptors for various pathogens were expressed on the surface of the platelet, enabling platelet to directly recognize and respond to invading viruses [35, 36]. The research from Milka Koupenova.et al. displayed that influenza virus during acute influenza infection can potentially invade into blood and become engulfed by platelets, then digestion of the virus was observed in platelet [37]. Seok-Joo Kim .et al. also observed a marked increase in platelet accumulation and the formation of large platelet aggregates within the lung vasculature following influenza A virus infection [38]. Influenza virus infection lead to the activation and consumption of platelet, contributing to the obvious drop of platelet count in peripheral blood as observed in the present study. PDW had been disclosed to be a more specific indicator of platelet reactivity than MPV [39, 40], an elevated PDW reflects increasing platelet destruction and variations in the size of newly formed immature platelets [41]. Being coincident with the previous studies, our research showed that the level of PDW rather than MPV rose significantly in the non-severe group for children with influenza (A and B), compared to the healthy control.

Additionally, unlike the trend of non-severe group, the severely infected ones exhibit a significant increase in the platelet count and a marked drop in the PDW. The main reason for this difference may be the different degree of systemic inflamma-tory response between severe and non-severe group. Satoshi Nishimura.et al. found that IL-1α could induce a much rapid platelet count increase into the vessel by a new way named “megakaryocyte rupture” in response to inflammatory stimuli or acute platelet requirement for platelet recovery as a host defense. Different to the traditional thrombopoiesis occurring via proplatelet formation (PPF) in the presence of thrombopoietin (TPO),the platelets release from megakaryocyte rupture appeared to a much more number and display more spheroid just liked mature platelets while PPF-dependent platelets were longitudinal and immature [42]. There are several studies have showed that influenza patients displayed markedly increased expression of IL-1α, and the expression level positively correlate with the severity of influenza infection, as well as the elevation of platelet count [42–45]. Notably, our earlier study has indicated that severe group of children with influenza expressed much higher levels of CRP and SAA, indicating more severe inflammatory response [18]. The present findings indirectly mirror the previous findings from a clinical point of view, this would also explain why the level of PDW decreased significantly in the severe group for children with influenza. The above results were similar to our study and confirmed that the change of platelet count and PDW may reflect the severity of influenza infection.

The limitations of this report included that, as a single-center study, the characteristics of enrolled children may not be representative, multi-center studies are needed to confirm our findings. In addition, the critically severe patients were not involved in this study, which would affect our conclusions, we would continue to collect more critically severe participants in future studies.

## Conclusions

In conclusion, there were several differences in clinical symptoms and laboratory indicators among children with influenza A and B. The change of platelet count and PDW may reflect the severity of influenza infection. Therefore, this present study suggests that routine blood examination, being easy to perform and inexpensive, in children with influenza should be performed to provide an important laboratory basis for accuracy diagnosis and severity assessment.

## Data Availability

The datasets used and analysed during the current study are available from the corresponding author on reasonable request.
